# Risks to patient safety associated with implementation of electronic applications for medication management in ambulatory care - a systematic review

**DOI:** 10.1186/1472-6947-13-133

**Published:** 2013-12-05

**Authors:** Cheryl LL Carling, Ingvild Kirkehei, Therese Kristine Dalsbø, Elizabeth Paulsen

**Affiliations:** 1 The Norwegian Knowledge Centre for the Health Services, PO Box 7004, St. Olavsplass, 0130 Oslo, Norway

**Keywords:** Electronic interventions, Medication management, Computer prescribing, Prescribing errors, Patient safety

## Abstract

**Background:**

The objective was to find evidence to substantiate assertions that electronic applications for medication management in ambulatory care (electronic prescribing, clinical decision support (CDSS), electronic health record, and computer generated paper prescriptions), while intended to reduce prescribing errors, can themselves result in errors that might harm patients or increase risks to patient safety.

**Methods:**

Because a scoping search for adverse events in randomized controlled trials (RCTs) yielded few relevant results, we systematically searched nine databases, including MEDLINE, EMBASE, and The Cochrane Database of Systematic Reviews for systematic reviews and studies of a wide variety of designs that reported on implementation of the interventions. Studies that had safety and adverse events as outcomes, monitored for them, reported anecdotally adverse events or other events that might indicate a threat to patient safety were included.

**Results:**

We found no systematic reviews that examined adverse events or patient harm caused by organizational interventions. Of the 4056 titles and abstracts screened, 176 full-text articles were assessed for inclusion. Sixty-one studies with appropriate interventions, settings and participants but without patient safety, adverse event outcomes or monitoring for risks were excluded, along with 77 other non-eligible studies. Eighteen randomized controlled trials (RCTs), 5 non-randomized controlled trials (non-R,CTs) and 15 observational studies were included. The most common electronic intervention studied was CDSS and the most frequent clinical area was cardio-vascular, including anti-coagulants. No RCTS or non-R,CTS reported adverse event. Adverse events reported in observational studies occurred less frequently after implementation of CDSS. One RCT and one observational study reported an increase in problematic prescriptions with electronic prescribing

**Conclusions:**

The safety implications of electronic medication management in ambulatory care have not been established with results from studies included in this systematic review. Only a minority of studies that investigated these interventions included threats to patients’ safety as outcomes or monitored for adverse events. It is therefore not surprising that we found little evidence to substantiate fears of new risks to patient safety with their implementation. More research is needed to focus on the draw-backs and negative outcomes that implementation of these interventions might introduce.

## Background

E-prescribing or the e-prescription (e-Rx) is defined in the Center for Medicare and Medicaid Services’ “final rule” as “…. the transmission, using electronic media, of prescription or prescription-related information between a prescriber, dispenser, pharmacy benefit manager, or health plan, either directly or through an intermediary, including an e-prescribing network” [1]. The implementation of e-Rx has been heralded as a remedy for problems associated with paper-based prescribing that can result in suboptimal or harmful outcomes for patients (e.g. [2]). The world’s first e-Rx for an ambulatory patient was transmitted from a physician’s office to a community pharmacy in Sweden in 1983 and by the end of 2008 it was estimated that about 80% of all new prescriptions in ambulatory care in Sweden were e-Rxs [3]. Other implementations of e-prescribing are in the USA [4] and Norway [5]. Canada is hoping “to make e-prescribing a reality by 2015” [6]. In addition to the e-Rx, the electronic health record (EHR) and clinical decision support systems (CDSS), both e-applications that can support clinicians’ prescribing activities, have been widely implemented and are regarded as important means to reduce the number of errors in medication management. CDSSs are implemented with the intention of improving clinical decision making in diagnosis and treatment [7], often for the purpose of changing provider behaviour (e.g. [8]); or supporting rational decision making and/or accurate use of clinical logarithms [9,10].

Systems with advanced clinical decision support integrated with the EHR have been acclaimed and supported by research as the interventions most likely to reduce adverse events in medication management; e.g. drug-drug interactions, allergic reactions and faulty dose calculations; to support adherence to best practice and to best support clinical decision making e.g. [11-16].

### Why there is a need for a systematic review

Despite the above accolades, concern has been expressed that digital applications might facilitate or provide new opportunities for prescribing errors and adverse events (AEs) or adverse drug events (ADEs) (e.g. [2,17]. Errors have been attributed to those that occur during input and access of information, and to communication and coordination processes between coupled applications or between application and end-user [18]. Examples of errors are faulty algorithms for dose calculation; wrong default dosing or route; user-interface facilitated errors; fragmentation errors due to faulty integration between applications; programming errors; as well as “false expectations” of clinicians who rely too whole-heartedly on the system’s capabilities [13,17,19-21].

Our scoping literature search did not identify systematic reviews or randomized studies in the field that might substantiate these assertions. One review that classified outcome type found only four of 30 included studies (1950 to March 31, 2006) that assessed safety [22]. Another [23] lists adverse drug events (ADEs) and deaths as patient outcomes and identifies the studies in which AEs and monitoring for AEs were specified *à priori*. Although we found relevant reviews that explored the effectiveness and efficiency of organizational interventions (e.g. CDSS) on improving process or patient outcomes (e.g. [15], to our knowledge, there is no earlier systematic review of which the main objective is to identify adverse events or harms to patients caused by organizational interventions.

### Objectives

The objective for this review was to gather evidence that might substantiate assertions that implementation of e-interventions for medication management in ambulatory care introduces new risks to patient safety.

## Methods

### Criteria for considering studies for this review

#### Types of studies eligible for this review

There is little guidance on methods to conduct systematic reviews to answer questions about adverse effects, adverse events or patient harms albeit a few methodological reviews explore the challenges (e.g. [24,25]) and the merits of including various study designs (e.g. [26,27]).

Randomized controlled studies are underused in evaluating medical informatics interventions because of constraints, e.g. “(1) ethical considerations, (2) difficulty of randomizing subjects, (3) difficulty to randomize by locations ......., (4) small available sample size” [28]. In line with this, our preliminary scoping search failed to locate any randomized controlled trials (RCTs) of e-applications in medication management in ambulatory care of which the object was to discover or measure adverse events or risks to patient safety. There is also convincing evidence that harms or adverse events are inadequately reported by randomized trials [29]. It is proposed that information on harms from both long-term prospective studies as well as RCTs should be used in identifying harms [26].

Based on the above and observations of other authors [30-34], we decided to include a wide assortment of study designs: randomized controlled trials (RCTs); non-randomized trials with concurrent control (n-RCTs); controlled trials; uncontrolled or non-concurrent controlled trials; cohort studies; cross-sectional studies and other observational designs.

### Interventions, settings and participants

#### Interventions

The intervention had to comprise at least one of the following e-applications:

– electronic transmission of individual patients’ prescriptions to a pharmacy or digital prescription repository accessible to community pharmacies (e-Rx),

– computer generation of paper prescriptions from prescriber’s computer (CGPRx) meant to be retrieved at the community pharmacy by the patient or her care giver,

or

– at least one of the following e-applications used at point-of-care and in real time during medication management, with or without e-Rx or CGPRx,: digital clinical decision support systems (CDSS). electronic health record (EHR).

In the research and discussion literature, the terms “e-prescribing” and “computerized prescribing” are sometimes interchangeably used to label medication management systems that might comprise one or more IT applications. For example, a system designated as “electronic prescribing” used “electronic prescribing software” with “clinical decision support” to generate either a paper or a digital prescription [35]. Occasionally, it is not clear exactly which e-applications are operative when the label “e-prescribing” is used without more specification, e.g. [36].

In this review we define an electronic prescription (e-Rx) as the digital message sent by an authorized prescriber to a pharmacy or a digital prescription repository that is accessible to community pharmacies. A telefax to a pharmacy is one of the simplest forms of an e-prescription. E-prescribing is the act of using electronic information processing, usually the personal computer (pc), to generate the e-prescription.

In this review we define CDSS as electronic access, via stationary or portable computer, including appliances like Palm Pilot, to any information that supports clinicians’ prescribing or monitoring of a patient’s medication regime at point-of-care [37] from simple drop-down formulary lists (so-called passive decision support) to sophisticated systems that access patient-specific data from the EHR and e.g. use clinical algorithms to compare prescribing to a knowledge base, generate patient-specific recommendations or trigger tailored e-alerts to the prescriber in real time, i.e. at the time of medication management (so-called active decision support). In summation, CDSS in this review includes all e-applications that support selection or dosing regimens of medications or monitoring of parameters relevant for medication management (therapeutic drug monitoring). Examples of CDSS are electronic reminders, alerts, medication pick-lists, formularies, dose calculators, medication regime management suggestions or guideline presentation. A CDSS can be stand-alone or coupled to the EHR and/or an e-prescribing application.

We use the Health Information Management System’s Society’s definition of the EHR [38]:

“The Electronic Health Record (EHR) is a longitudinal electronic record of patient health information generated by one or more encounters in any care delivery setting.”

#### Setting and participants

We included publications written in English or a Scandinavian language published after 1994 that reported effects of or observations associated with the implementation of the e-applications under consideration and implemented in ambulatory care, (i.e. doctors’ offices, out-patient clinics, emergency rooms, well-baby clinics), targeted at the clinician, (i.e. health practitioners authorized to prescribe medications, i.e. nurses, nurse practitioners, physician assistants, and physician) for the purpose of medication management in the broad sense, i.e. new prescribing (including vaccinations) or medication regime adjustments: or activities performed for the purpose of evaluation and modification of patients’ therapeutic pharmacological regime, e.g. monitoring of relevant physiological and drug parameters. Eligible participants were doctors or nurses licensed or otherwise eligible to prescribe medications in the setting.

#### Outcomes

We included studies that reported quantifiable objective outcome measures and comprised:

– Harms or threats to patient safety defined as outcomes in the study methods section.

– Any adverse events, as defined by the investigators.

– Anecdotal reports of events that could signify threats to patient safety.

Outcomes could be:

– Process of care: e.g. unsafe prescribing.

– Patient outcomes: e.g. mortality; morbidity, e.g. major bleeds, thrombotic events; adverse drug events (ADEs): e.g. unintended effects of drugs including drug-drug and drug-disease interactions; clinical course e.g. admission to specialist facility, emergency room visit.

– Technological outcomes: e.g. errors in transmission, errors in algorithm programming.

Criteria for exclusion were:

– studies performed in an in-patient setting or a combination of in- and out-patient settings where outcomes were not differentiated according to this classification

– primary endpoints were subjective measures (patients’ or clinicians’)

– studies where non-e-interventions were mixed with e-interventions and outcomes were not differentiated according to this classification

– studies where medication management outcomes were part of a bundle that included non-medication management outcomes and outcomes were not reported according to this classification

– the intervention was not delivered at point-of-care, in real-time and for an individual patient

– the intervention was only a direct order entry of a medication to be administered by clinic staff, except for immunizations

– simulated interventions

### Strategy for identification of eligible studies

#### Electronic searches

We searched for systematic reviews, randomized controlled trials, non-randomized controlled trials and observational studies that investigated the use of e-applications in medication management in ambulatory care and reported quantifiable outcomes. We performed systematic searches for literature published from 1995 to 2012 in the following databases: Ovid MEDLINE, Ovid EMBASE, Ovid British Nursing Index, ISI Social Science/Science Citation Index, Cochrane Database of Systematic Reviews, Cochrane CENTRAL, Database of Abstracts of Reviews of Effects (DARE), Health Technology Assessment Database (HTA), PubMed and SveMed. The first search was performed in September 2008 and was updated three times. The last search was performed August 8, 2012 (Additional file 1: Table S1).

The search strategy consisted of subject headings and text words covering the concepts of electronic prescriptions and decision support as well as related concepts like medical informatics and electronic patient journal (EPJ) (combined with “prescriptions/medication”). These search terms were combined with terms for ambulatory care/primary health care or patient safety. We also performed a supplementary broad search in the Cochrane Central EPOC register to retrieve all studies with the subject heading “Information systems” (including subheadings).

#### Hand-searching

Hand searches were performed by one investigator (CLC) for relevant studies, i.e. inspection of lists of included studies from relevant systematic reviews (Additional file 2: Table S2) and reference lists from discussion papers and relevant studies.

#### Study evaluation for inclusion

Preliminary screening of the identified publications from the first two and fourth systematic searches was carried out independently by pairs of researchers (EJP & CLC and CLC & TKD, respectively) who evaluated relevance by reading abstracts and when no abstract was available, based on the title or full text version. One reviewer (CLC) evaluated titles and abstracts found on the third systematic search. We retrieved full-text articles for all studies judged as relevant. Two researchers (CLC & TKD) independently evaluated studies in their full-text version for inclusion and we discussed and agreed upon inclusion of the studies.

#### Data extraction and management

Two authors (CLC & TKD) extracted estimates and funding data from randomized controlled trials. There was no disagreement between reviewers. One author (CLC) entered these estimates plus the following data into SPSS data bases for all studies: study design; setting; participants; objectives; intervention; comparator; and any relevant observed outcomes. For all studies, we recorded anecdotal reports of unexpected or adverse events that were reported to have or in the investigators’ or authors’ opinion, could have signified or caused compromises to patient safety. Study quality of the included RCTs was assessed independently, discussed and agreed upon by two investigators (CLC & TKD).

#### Quality assessment

Two reviewers (CLC, TKD) independently assessed, discussed and agreed upon the quality of the RCTs using a supplemented version of the EPOC checklist [39] (Additional file 3: Table S3). We did not assess quality for the other study designs.

## Results

### Results of search and selection process

**Figure 1:**
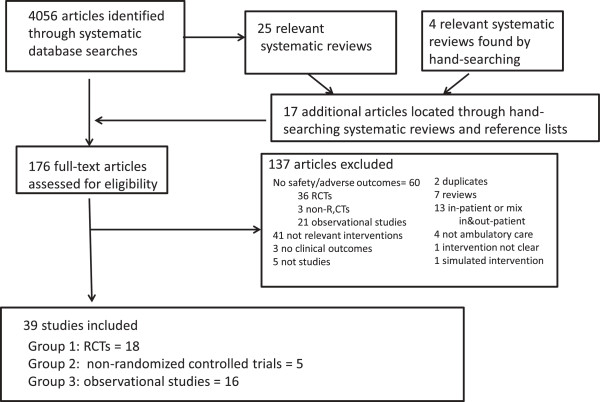
Flow chart of search and selection process.

We obtained full-text articles for 159 publications by screening titles and abstracts of the 3941 unique studies identified by systematic searches, plus another 17 studies identified from hand searching reference lists in relevant studies and systematic reviews, giving a total of 176 potential studies for inclusion. We did not find any systematic reviews that examined adverse events or harms to patients caused by organizational interventions. One-hundred thirty-seven of these studies were excluded (Figure 1). Thirty-six of these excluded studies were RCTs with relevant interventions, participants and settings but either had no outcomes that were directly related to risks to patient safety, did not report any adverse events or did not monitor for them. As well, 3 non-R,CTs and 21 observational studies were excluded for this reason (Figure 1; Additional file 4: Table S4; Additional file 5: Table S5; Additional file 6: Table S6).

We included 39 individual studies, comprising three study design groups: RCTs (n = 18); non-R,CTs (n = 5); and observational studies (n = 16) (Additional file 7: Table S7; Additional file 8: Table S8; Additional file 9: Table S9).

### Randomized controlled trials (n = 18)

#### Description of included RCTS

Eighteen randomized controlled trials specified outcomes related to safety, e.g. “unsafe prescribing”, “error rate”, had adverse events as outcomes, or specified *à priori* that monitoring for adverse events was planned [35,40-56]. Settings for these 18 RCTs were doctors’ offices (n = 9; 50%), hospital out-patient (n = 6; 33%), both doctors’ offices and hospital out-patient clinics (n = 1; 6%), and emergency room (n = 2; 11%) (Additional file 10: Table S10).

E-Rx was investigated in only one RCT, a pragmatic trial [35] along with user-initiated CDSS and an e-application to print paper prescriptions (eGPP). All RCTs investigated CDSS, whereof the CDSS was system-initiated in 16 studies and user-initiated in 2 studies. Uptake of intervention, adherence or non-override of CDSS advice was poor in six of the eight studies that reported this. In all the 10 studies where it was apparent that a EHR was in operation, it was, or appeared to be, in the same digital environment as the CDSS application. In all, eGPP was mentioned to be in use in two RCTs [35,40].

The most frequent drug classes and clinical areas studied were anti-coagulants (5 studies) [42-44,49,56]; other cardio-vascular or heart disease management (n = 4) [40,46,48,54], and elderly patients (n = 4) [45,50-52]. Most of the RCTs were funded by non-industry grants or similar. We found a risk of funding bias in only one study [43]. Quality assessment of included RCTs is provided in Additional file 11: Table S11.

#### Outcomes for RCTs

Eleven studies had adverse events or serious negative results as main outcomes [40,43,44,46-49,51,54-56]. One of these showed a statistically higher estimated risk of “cardiovascular events” (based on surrogate markers) when CDSS was compared with a risk chart but no significant differences when the compared was with traditional dosing. However, no actual adverse events were reported, despite explicit monitoring for them [48]. Reduction in risk of injury was statistically significant in [51] and there were statistically significant improvements for heart failure patients’ outcomes in [40] and three of seven outcomes for asthma patients [47]. In the remaining studies, there were no significant differences or so low event incidence that calculation of significance was meaningless.

Problematic prescribing or prescriptions (e.g. unsafe/risky prescribing, prescribing errors, unclear prescription) were the outcomes of seven studies [35,41,42,45,50,52,53]. Significantly better results for the intervention group were found in five studies [41,42,45,50,52,53], significantly worse results were found in one study [35] and non-significant differences in the remaining study.

#### Non-randomized controlled studies (n = 5)

Of the five included studies, three investigated CDSS [57-59], one investigated computer-generated paper prescriptions [11], and one investigated e-prescribing [60]. All studies investigated prescribing errors and one included potential adverse drug events as an outcome [11]. No study found any adverse events and other outcomes were either better or unchanged in all studies. One study reported that all errors in the CDSS intervention group were concordant with override of decision support advice [58] (Additional file 12: Table S12).

#### Observational studies (n = 16)

Fifteen studies had the following pre-specified outcomes: faulty, risky, unclear prescriptions or prescriptions with errors (n = 11) [61-71], unclaimed e-prescriptions (n = 2) [72,73], and adverse drug events (ADEs) (n = 3) [62,74,75]. We also included one retrospective series study with main outcome of overriding alerts because the authors reported that the only three ADEs that occurred in the study were consistent with alert non-compliance [76]. In addition, ADEs were anecdotally reported in [63]. Of the four studies that reported before-and-after ADEs, there were fewer ADEs after implementation of computer generated paper prescriptions in one study [74] and better or unchanged incidence after implementation of CDSS in two studies [62,75]. Three adverse events were related to interacting drug prescriptions where the prescriber did not comply with CDSS advice [77]. E-prescribing was evaluated in three studies [61,72,73]. Only one study [61] showed a result in disfavor of the e-intervention, in that more clarifications were needed by pharmacists for e-prescriptions than for non-e-prescriptions. Two of these studies reported that some patients who did not retrieve their e-prescribed drugs at the pharmacy said this was because they had no paper prescription to remind them [72,73]. In one of these [73], 33% of the unclaimed prescriptions were for “essential drugs. User-interface problems were reported as the cause of prescribing error in two studies. A non-controlled before-and-after study found errors in CDSS pick-lists and wrong information concerning available formulations and duration of treatment [74]. A retrospective survey reported high rates of discrepancies between free-text and check-off fields on e-prescriptions, of which investigators assessed that 16% of discrepancies could lead to hospital admission or death [67] (Additional file 13: Table S13).

## Discussion

Randomized and non-randomized controlled trials did not show any adverse patient events. The incidence of adverse events/adverse drug events did not increase post-intervention in observational studies. The quality of prescriptions was significantly worse in only one RCT [35] and one pre-post observational study [61]. One non-R,CT reported all prescribing errors were concordant with override of the CDSS advice [58].

From the studies assessed in this review, it appears that observational studies give us most insight into potential causes of adverse events or potential for patient harm. User-interface, faulty programming and erroneous information in the CDSS application were problems that lead to erroneous prescribing [67,74]. Adverse events were found to be concordant with non-adherence to CDSS suggestions [77]. Some patients forgot to pick up e-prescribed drugs for important medications because they had no paper reminder [72,73].

Contrary to the “false expectation” hypothesis, presented in the introduction, that excessive reliance on or confidence in an e-application for medication management would facilitate errors or adverse events, there was low compliance with CDSS advice among the eight RCTs that reported this, consistent with other’s findings [76,78] and all adverse events in two studies were concordant with CDSS non-compliance [58,77]. This may be due to professionals’ skepticism or fear of loss of autonomy, e.g. [79].

### Limitations to the review

As far as we are aware, this is first systematic review to focus on the risks and adverse outcomes of e-interventions for medication management in ambulatory care settings. Its findings, however, are limited, and should be interpreted with caution. We did not find randomized or non-randomized studies of which the main objective was to evaluate the safety, or, conversely, the risks associated with implementation of e-interventions for medication management in ambulatory care. Only one observational study focused on “unintended consequences” [67]. Although we used an all-inclusive approach, scrutinizing studies of almost any design where an e-intervention was used, only a minority of the individual studies we initially assessed for inclusion were concerned with safety issues at all. Sixty-five percent of the 99 studies with appropriate interventions, settings and participants that we assessed did not investigate, monitor for or mention anecdotally adverse events or risks to patient safety and therefore were not included in this review. Sixty-six per cent (36) of otherwise eligible RCTs were excluded for this reason, leaving only 18 RCTs for inclusion. Unfortunately, even where adverse events are not the main study outcomes, RCTs are apparently often not powered to reveal seldom or beforehand unknown outcomes or do not have a long-enough or appropriate follow-up. Even non-significant findings of adverse events may indeed have clinical significance, especially if they were made available for meta-analysis [80]. Publication bias [81,82] and funding bias might also contribute to the dearth of published studies investigating or reporting adverse events or patient safety outcomes.

### Weaknesses of the review

It is possible that we did not locate all relevant studies in our searches. Firstly, of studies found in the third systematic search, screening of titles and abstracts, and selection of studies for which to obtain full-text publications was conducted by only one researcher. Secondly, harm or adverse effects are often inadequately reported in the research literature, poorly indexed in the medical databases and might be difficult to identify. Therefore, searching for adverse effects requires highly sensitive search filters with several different subject headings and text words [25,83]. It is difficult to create effective adverse effects search filters; therefore better reporting and indexing of adverse effects is required [25]. To overcome this issue, we made a parallel search, not restricted to adverse effects, but for studies performed in ambulatory care settings. However, many studies do not have adequate descriptions of the clinical research setting in their abstracts and there is inconsistent use of terminology [84]. There are constantly new studies being published on this topic and it is possible that updates of this review might alter conclusions.

## Conclusions

As so few studies were looking for them, it is not surprising that we found little evidence that might substantiate assertions that implementation of e-applications in medication management in ambulatory settings causes or introduces new untoward risks, adverse events or harms to patients. Until more evidence is sought and presented to disprove the apparently prevailing assumption of the null hypothesis that the e-interventions do not introduce risk, it is premature to conclude that we can fail to reject it.

### Implications for practice

The safety implications of e-medication management in primary care have not been established with results from existing studies found in this systematic review. Regarding, however, the substantial investment necessary to implement e-technologies for medication management and the potential for improving decision making and quality of care, it would be prudent for health care providers to consider attributes of interventions that might have been shown to induce uptake of the intervention or reduce override of CDSS advice (e.g. [85]). For example, there is some evidence that user-initiation of the e-application and forced justification of overrides might facilitate uptake.

Paper reminders for prescription redemption might help patients remember to claim their e-prescribed medications at the pharmacy [72,73]. Faulty programming of CDSS might facilitate patient harm [74] while programming built-in constraints into e-applications might eliminate potentially dangerous rule violations and discrepancies [67].

### Implications for research and reporting of research

Well-designed RCTs are needed to investigate hypothesized untoward and adverse effects of e-interventions for medication management in ambulatory care. It has been suggested that a before and after clustered RCT utilizing an incomplete block design is the optimal approach to evaluate CDSS in primary care [86].

While harm outcomes may be the most sensitive and valid endpoints to evaluate the implementation of systems that effect safety, it is unethical in prospective studies to let the consequences of professional or system errors reach the patient. As well, in the real world, most prescribing errors with potential to cause harm are intercepted before they reach the patient. Surrogate endpoints established as patient safety indicators might be employed. Factors instrumental to observed errors should be determined and alleviated. Valid and reliable methods to assess the potential severity of errors and other surrogate end-points should be developed.

Investigators conducting studies of the effect of organizational interventions should power their studies and length of follow-up to capture rare, unintended and unsuspected events and publish these findings regardless of statistical significance. Even anecdotal reports of observed risks to patient safety and adverse events can inform future study hypotheses and design.

We encourage investigators to improve reporting quality with use of the CONSORT statement, especially the extended version for cluster randomized trials [87]. In reporting studies of e-interventions for medications management, authors should be explicit about which specific applications were studied, their features and implementation strategy, and should consider using standard definitions. As well, clearer descriptions of the study methodology and more consistent use of study design labels would be helpful to perform a more efficient search for observational studies [88].

## Competing interests

The authors declare that they have no financial, professional or personal competing interests in association with the findings of this review. All funding was provided by the Norwegian Knowledge Centre for the Health Services.

## Authors’ contributions

CLC takes responsibility for the integrity of the work as a whole, from inception to published review, as the principle researcher, planned and coordinated the review, developed the literature search strategy, selected articles, extracted data, evaluated study quality, wrote the first draft of the paper and revisions; IK developed the systematic search strategy and search algorithm plus revisions, conducted the searches and contributed to writing the first draft; TKD selected studies for inclusion, extracted data and evaluated study quality; contributed to revisions of the manuscript; EJP participated in article screening and selection. All authors read and approved the final version of the manuscript.

## Pre-publication history

The pre-publication history for this paper can be accessed here:

http://www.biomedcentral.com/1472-6947/13/133/prepub

## Supplementary Material

Additional file 1: Table S1 Search strategy. Click here for file

Additional file 2: Table S2 Systematic reviews screened for eligible studies. Click here for file

Additional file 3: Table S3 Explanation of quality assessment for included RCTs. Click here for file

Additional file 4: Table S4 Excluded randomized controlled trials (RCTs) citations. Click here for file

Additional file 5: Table S5 Excluded non-randomized controlled studies (non-R,CTs) citations. Click here for file

Additional file 6: Table S6 Excluded observational studies citations. Click here for file

Additional file 7: Table S7 Included randomized controlled trials (RCTs) citations. Click here for file

Additional file 8: Table S8 Included non-randomized controlled studies citations. Click here for file

Additional file 9: Table S9 Included observational studies citations. Click here for file

Additional file 10: Table S10 Included randomized controlled studies (RCTs); characteristics and outcomes. Click here for file

Additional file 11: Table S11 Quality assessment of included RCTs. Click here for file

Additional file 12: Table S12 Included non-randomized controlled trials (non-R,CTs); characteristics and outcomes. Click here for file

Additional file 13: Table S13 Included observational studies; characteristics and outcomes. Click here for file
