# Supplementary material for: Risks to patient safety associated with implementation of electronic applications for medication management in ambulatory care - a systematic review
Source: BMC Med Inform Decis Mak. 2013 Dec 5;13:133. doi: 10.1186/1472-6947-13-133 (PMC3913838; doi:10.1186/1472-6947-13-133)
Supplement: Additional file 1: Table S1 — Search strategy. [file 1472-6947-13-133-S1.pdf]

Table S2

Systematic reviews screened for eligible studies

n = 29

Arnold SR, Straus SE: **Interventions to improve antibiotic prescribing practices in ambulatory care.** *Cochrane Database Syst Rev* 2005, (4):CD003539.
Balas EA, Krishna S, Kretschmer RA, Cheek TR, Lobach DF, Boren SA: **Computerized knowledge management in diabetes care.** *Med Care* 2004, **42**:610-621.
Bennett JW, Glasziou PP: **Computerised reminders and feedback in medication management: A systematic review of randomised controlled trials.** *Med J Aust* 2003, **178**:217-222.
Bryan C, Boren SA: **The use and effectiveness of electronic clinical decision support tools in the ambulatory/primary care setting: a systematic review of the literature.** *Inform Prim Care* 2008, **16**:79-91.
Chatellier G, Colombet I, Degoulet P: **An overview of the effect of computer-assisted management of anticoagulant therapy on the quality of anticoagulation.** *Int J Med Inf* 1998, **49**:311-320.
Chatellier G, Colombet I, Degoulet P: **Computer-adjusted dosage of anticoagulant therapy improves the quality of anticoagulation.** *Medinfo* 1998, **9 Pt 2**:819-823.
Conroy S, Sweis D, Planner C, Yeung V, Collier J, Haines L, Wong IC: **Interventions to reduce dosing errors in children: a systematic review of the literature.** *Drug Saf* 2007, **30**:1111-1125.
Costa BM, Fitzgerald KJ, Jones KM, Dunning Am T: **Effectiveness of IT-based diabetes management interventions: a review of the literature.** *BMC Fam Pract* 2009, **10**:72.
Durieux P, Colombet I, Niès J, Walton RT, Rajeswaran A, Rége-Walther M, Harvey E, Burnand B: **Computerized advice on drug dosage to improve prescribing practice.** *Cochrane Database Syst Rev* 2008, (3):CD002894.
Eslami S, Abu-Hanna A, de Keizer NF: **Evaluation of outpatient computerized physician medication order entry systems: a systematic review.** *J Am Med Inform Assoc* 2007, **14**:400-406.
Eslami S, Abu-Hanna A, de Jonge E, de Keizer NF: **Tight glycemic control and computerized decision-support systems: a systematic review.** *Intensive Care Med* 2009, **35**:1505-1517.
Fitzmaurice DA, Hobbs FD, Delaney BC, Wilson S, McManus R: **Review of computerized decision support systems for oral anticoagulation management.** *Br J Haematol* 1998, **102**:907-909.
Garg AX, Adhikari NKJ, McDonald H, Rosas-Arellano MP, Devereaux PJ, Beyene J, Sam J, Haynes RB: **Effects of computerized clinical decision support systems on practitioner performance and patient outcomes: A systematic review.** *JAMA* 2005, **293**:1223-1238.
Hayward GL, Parnes AJ, Simon SR: **Using health information technology to improve drug monitoring: a systematic review.** *Pharmacoepidemiol Drug Saf* 2009, **18**:1232-1237.

Hider P: **Electronic prescribing. A critical appraisal of the literature**. *NZHTA Report* 2002, **5**(2).

Hunt TL, Haynes RB, Hanna SE, Smith K: **Effects of computer-based clinical decision support systems on physician perfomance and patient outcomes: a systematic review.** JAMA 1998, **280**:1339-1346.

Jamal A, McKenzie K, Clark M: **The impact of health information technology on the quality of medical and health care: a systematic review.** *HIM J* 2009, **38**:26-37.

Kastner M, Straus SE: **Clinical Decision Support Tools for Osteoporosis Disease Management: A Systematic Review of Randomized Controlled Trials.** *J Gen Intern Med* 2008, **23**:2095-2105.

Mitchell MD, Copelli F, Shawhughes L, Laskin M, Foght F, Gibson G, Young DS, Umscheid C, Williams K: *Point of care testing for monitoring anticoagulation therapy.* Philadelphia: Center for Evidence-based Practice (CEP); 2008.

Mollon B, Chong JJ, Holbrook AM, Sung M, Thabane L, Foster G: **Features predicting the success of computerized decision support for prescribing: a systematic review of randomized controlled trials.** *BMC Med Inf Decis Mak* 2009, **9**:11.

Montgomery AA, Fahey T: **A systematic review of the use of computers in the management of hypertension.** *J Epidemiol Community Health* 1998, **52**:520-525.

Pearson SA, Moxey A, Robertson J, Hains I, Williamson M, Reeve J, Newby D: **Do computerised clinical decision support systems for prescribing change practice? A systematic review of the literature (1990-2007).** *BMC Health Serv Res* 2009, **9**:154.

Schedlbauer A, Prasad V, Mulvaney C, Phansalkar S, Stanton W, Bates DW, Avery AJ: **What evidence supports the use of computerized alerts and prompts to improve clinicians' prescribing behavior?** *J Am Med Inform Assoc* 2009, **16**:531-538.

Shamliyan TA, Duval S, Du J, Kane RL: **Just what the doctor ordered. Review of the evidence of the impact of computerized physician order entry system on medication errors.** *Health Serv Res* 2008, **43**:32-53.

Sintchenko V, Magrabi F, Tipper S: **Are we measuring the right end-points? Variables that affect the impact of computerised decision support on patient outcomes: A systematic review.** *Med Inform Internet Med* 2007, **32**:225-240.

van der Sijs H, Aarts J, Vulto A, Berg M: **Overriding of Drug Safety Alerts in Computerized Physician Order Entry.** *J Am Med Inform Assoc* 2006, **13**:138-147.

Walton RT, Harvey E, Dovey S, Freemantle N: **Computerised advice on drug dosage to improve prescribing practice.** *Cochrane Database Syst Rev* 2001, (1):CD002894.

Wolfstadt JI, Gurwitz JH, Field TS, Lee M, Kalkar S, Wu W, Rochon PA: **The effect of computerized physician order entry with clinical decision support on the rates of adverse drug events: a systematic review.** *J Gen Intern Med* 2008, **23**:451-458.

Yourman L, Concato J, Agostini JV: **Use of computer decision support interventions to improve medication prescribing in older adults: A systematic review.** *Am J Geriatr Pharmacother* 2008, **6**:119-129.
