# Supplementary material for: Risks to patient safety associated with implementation of electronic applications for medication management in ambulatory care - a systematic review
Source: BMC Med Inform Decis Mak. 2013 Dec 5;13:133. doi: 10.1186/1472-6947-13-133 (PMC3913838; doi:10.1186/1472-6947-13-133)
Supplement: Additional file 2: Table S2 — Systematic reviews screened for eligible studies. [file 1472-6947-13-133-S2.pdf]

Table S3 Explanation of quality assessment for included RCTs

Method of protection against bias	Type of bias	Scoring
Allocation concealment with explicit randomization	Selection bias	If the unit of allocation was by institution, team or professional<br>- LOW RISK OF BIAS if any random process is described explicitly, e.g. the use of random number tables or coin flips and was done at the start of the study;<br>-HIGH RISK OF BIAS if non-random method was used;<br>- UNCLEAR RISK OF BIAS if the method is not described in the paper.<br><br>If the unit of allocation was by patient or episode of care, score<br>- LOW RISK OF BIAS if there was some form of centralized randomization scheme, an on-site computer system or sealed opaque envelopes were used;<br>-HIGH RISK OF BIAS if non-random method was used;<br>- UNCLEAR RISK OF BIAS if the method is not described in the paper.
Follow-up of units included (Practices &/or professionals &/or patients)	Attrition bias	- LOW RISK OF BIAS if outcome measures were obtained for 80-100% of units included;<br>-HIGH RISK OF BIAS if outcome measures were obtained for less than 80% of units included or if missing outcome data was likely to bias the results;<br>- UNCLEAR RISK OF BIAS if not described in the paper
Blinded assessment of primary outcomes	Detection bias	- LOW RISK OF BIAS if outcome the authors state that outcome variables were assess blindly or the outcome variables are objective, e.g. length of hospitalization, drug levels as assess by standardized tests measures were obtained for 80-100% units randomized or units of analysis:<br>-HIGH RISK OF BIAS if the outcomes were not assessed blindly<br>- UNCLEAR RISK OF BIAS if not reported in the paper
Baseline measurement of outcomes	Selection bias	- LOW RISK OF BIAS if patient or outcome measures were measured prior to the intervention and no substantial differences were present across study groups;<br>-HIGH RISK OF BIAS if there were differences at baseline in main outcome measures likely to undermine the post intervention differences;<br>- UNCLEAR RISK OF BIAS if baseline measures are not reported in the paper or it is unclear that baseline measures are substantially different across groups
Reliability of	Detection bias	- LOW RISK OF BIAS if manual data extraction was done by

reported primary outcome measures		two or more data extractors with reported at least 90% agreement or kappa greater than or equal to 0.8; OR automated system used to obtain outcome measures, e.g. electronic patient journal;<br>-HIGH RISK OF BIAS if manual data extraction used, done by only one person, or less than 90% agreement or kappa greater than or equal to 0.8 between two or more data extractors;<br>- UNCLEAR RISK OF BIAS if manual data capture and reliability is not reported
Protection against contamination	Performance bias	- LOW RISK OF BIAS if allocation was by community, institution or practice and it is unlikely that the controls received the intervention;<br>- HIGH RISK OF BIAS if professionals were allocated within a clinic or practice and it is likely that communication between experimental and control group professionals influenced professional behavior; or if patients within a practice were randomized and it is highly likely, because of the nature of the intervention, that the control group received the intervention (e.g. cross-over trials or if patients rather than professionals were randomized;<br>- UNCLEAR RISK OF BIAS if professionals were allocated within a clinic or practice and it is possible that communication between experimental and control group professionals could have had influenced professional behavior; or patients within professionals were allocated resulting in a low likelihood that exposure to the intervention while treating intervention patients might influence professional behavior in treating control patients, e.g. individually patient-tailored algorithms.
All outcomes reported	Reporting bias	- LOW RISK OF BIAS if all relevant outcomes in the methods section are reported in the results section;<br>- HIGH RISK OF BIAS if some important outcomes are omitted from the results;<br>- UNCLEAR RISK OF BIAS if outcomes are not specified in the methods section.
Declaration of interests and funding	Funding bias or conflict of interest	- NO RISK FOUND: Funding reported from non-commercial interests and no conflict of interest reported or detected by reviewers, e.g. due to employment or other position<br>- HIGH RISK OF BIAS: Industry funding reported; researcher(s) employed or funded by industry, or conflict of interest reported;<br>- UNCLEAR RISK OF BIAS: Funding source not reported; or unclear if funder could have interest in study results.
Adjustment for clustering when indicated	Threat to reliability of variance estimate	Self-explanatory; see table
