# Supplementary material for: Risks to patient safety associated with implementation of electronic applications for medication management in ambulatory care - a systematic review
Source: BMC Med Inform Decis Mak. 2013 Dec 5;13:133. doi: 10.1186/1472-6947-13-133 (PMC3913838; doi:10.1186/1472-6947-13-133)
Supplement: Additional file 3: Table S3 — Explanation of quality assessment for included RCTs. [file 1472-6947-13-133-S3.pdf]

Table S5

Excluded non-randomized controlled studies (non-R,CTs) citations

n = 3

**Frank 2004**	Frank O, Litt J, Beilby J: Opportunistic electronic reminders. Improving performance of preventive care in general practice. *Aust Fam Physician* 2004, 33:87-90.
**Herrin 2012**	Herrin J, da GB, Nicewander D, Fullerton C, Aponte P, Stanek G, Cowling T, Collinsworth A, Fleming NS, Ballard DJ: The effectiveness of implementing an electronic health record on diabetes care and outcomes. *Health Serv Res* 2012, 47:1522-1540.
**Tang 1999**	Tang PC, LaRosa MP, Newcomb C, Gorden SM: Measuring the effects of reminders for outpatient influenza immunizations at the point of clinical opportunity. *J Am Med Inform Assoc* 1999, 6:115-121.
