# Supplementary material for: Risks to patient safety associated with implementation of electronic applications for medication management in ambulatory care - a systematic review
Source: BMC Med Inform Decis Mak. 2013 Dec 5;13:133. doi: 10.1186/1472-6947-13-133 (PMC3913838; doi:10.1186/1472-6947-13-133)
Supplement: Additional file 4: Table S4 — Excluded randomized controlled trials (RCTs) citations. [file 1472-6947-13-133-S4.pdf]

Table S6

Excluded observational studies citations

n = 21

**Bassa 2005**	Bassa A, Del Val M, Cobos A, Torremade E, Bergonon S, Crespo C, Brosa M, Munio S, Espinosa C: Impact of a clinical decision support system on the management of patients with hypercholesterolemia in the primary healthcare setting. *Dis Manag Health Outcomes* 2005, 13:65-72.
**Bell 2010**	Bell LM, Grundmeier R, Localio R, Zorc J, Fiks AG, Zhang XM, Stephens B, Swietlik M, Guevara JP: Electronic health record-based decision support to improve asthma care: A cluster-randomized trial. *Pediatrics* 2010, 125:E770-E777.
**El-Kareh 2011**	El-Kareh RE, Gandhi TK, Poon EG, Newmark LP, Ungar J, Orav EJ, Sequist TD: Actionable reminders did not improve performance over passive reminders for overdue tests in the primary care setting. *JAMIA* 2011, 18:160-163.
**Fitzmaurice 1998**	Fitzmaurice DA, Hobbs FD, Murray ET: Primary care anticoagulant clinic management using computerized decision support and near patient international normalized ratio (INR) testing: routine data from a practice nurse-led clinic. *Fam Pract* 1998, 15:144-146.
**Garthwaite 2004**	Garthwaite EA, Will EJ, Bartlett C, Richardson D, Newstead CG: Patient-specific prompts in the cholesterol management of renal transplant outpatients: results and analysis of underperformance. *Transplantation* 2004, 78:1042-1047.
**Gilligan 2012**	Gilligan AM, Miller K, Mohney A, Montenegro C, Schwarz J, Warholak TL: Analysis of pharmacists' interventions on electronic versus traditional prescriptions in 2 community pharmacies. *Res Social Adm Pharm* 2012, 8:523-532.
**Isaac 2009**	Isaac T, Weissman JS, Davis RB, Massagli M, Cyrulik A, Sands DZ, Weingart SN: Overrides of medication alerts in ambulatory care. *Arch Intern Med* 2009, 169:305-311.
**Lee 2011**	Lee K. A pilot follow-up study on the use of a reminder system among patients with unsatisfactory control of diabetes mellitus in a Hong Kong public family medicine clinic. *HK Pract* 2011, 33:56-62.
**Linder 2007**	Linder JA, Ma J, Bates DW, Middleton B, Stafford RS: Electronic health record use and the quality of ambulatory care in the United States. *Arch Intern Med* 2007, 167:1400-1405.
**McMullin 2004**	McMullin ST, Lonergan TP, Rynearson CS, Doerr TD, Veregge PA, Scanlan ES: Impact of an evidence-based computerized decision support system on primary care prescription costs. *Ann Fam Med* 2004, 2:494-498.
**Michelis 2011**	Michelis KC, Hassouna B, Owlia M, Kelahan L, Young HA, Choi BG: Effect of electronic prescription on attainment of cholesterol goals. *Clin Cardiol* 2011, 34:254-260.
**Montori 2002**	Montori VM, Dinneen SF, Gorman CA, Zimmerman BR, Rizza RA, Bjornsen SS, Green EM, Bryant SC, Smith SA: The impact of planned care and a diabetes electronic management system on community-based diabetes care - The Mayo Health System Diabetes Translation Project. *Diabetes Care* 2002, 25:1952-1957.
**Nicolucci 2008**	Nicolucci A, Allotta G, Allegra G, Cordaro G, D'Agati F, Di Benedetto A, Di Mauro M, Fulantelli M, Garofalo A, Giacchetto C et al.: Five-year impact of a continuous quality improvement effort implemented by a network of diabetes outpatient clinics. *Diabetes*

	Care 2008,57-62.
**Pearce 2010**	Pearce DD, Opperman JM: Electronic medical record reduces HIV medication refill response time and emergency refills in a Latino community clinic. *Int J STD AIDS* 2010, 21:184-186.
**Ross 2005**	Ross SM, Papshev D, Murphy EL, Sternberg DJ, Taylor J, Barg R: Effects of electronic prescribing on formulary compliance and generic drug utilization in the ambulatory care setting: a retrospective analysis of administrative claims data. *J Manage Care Pharm* 2005, 11:410-415.
**Shapiro 2011**	Shapiro A, Gracy D, Quinones W, Applebaum J, Sarmiento A: Putting guidelines into practice: improving documentation of pediatric asthma management using a decision-making tool. *Arch Pediatr Adolesc Med* 2011, 165:412-418.
**Shiffman 2000**	Shiffman RN, Freudigman M, Brandt CA, Liaw Y, Navedo DD: A guideline implementation system using handheld computers for office management of asthma: effects on adherence and patient outcomes. *Pediatrics* 2000, 105:767-773.
**Simon 2006**	Simon SR, Smith DH, Feldstein AC, Perrin N, Yang X, Zhou Y, Platt R, Soumerai SB: Computerized prescribing alerts and group academic detailing to reduce the use of potentially inappropriate medications in older people. *J Am Geriatr Soc* 2006, 54:963-968.
**Smith 2006**	Smith DH, Perrin N, Feldstein A, Yang X, Kuang D, Simon SR, Sittig DF, Platt R, Soumerai SB: The impact of prescribing safety alerts for elderly persons in an electronic medical record: an interrupted time series evaluation. *Arch Intern Med* 2006, 166:1098-1104.
**Subramanian 2004**	Subramanian U, Fihn SD, Weinberger M, Plue L, Smith FE, Udris EM, McDonell MB, Eckert GJ, Temkit M'H, Zhou XH et al.: A controlled trial of including symptom data in computer-based care suggestions for managing patients with chronic heart failure. *Am J Med* 2004, 116:375-384.
**Tamblyn 2008**	Tamblyn R, Huang A, Taylor L, Kawasumi Y, Bartlett G, Grad R, Jacques A, Dawes M, Abrahamowicz M, Perreault R et al.: A randomized trial of the effectiveness of on-demand versus computer-triggered drug decision support in primary care. *J Am Med Inform Assoc* 2008, 15:430-438.
