# Supplementary material for: Risks to patient safety associated with implementation of electronic applications for medication management in ambulatory care - a systematic review
Source: BMC Med Inform Decis Mak. 2013 Dec 5;13:133. doi: 10.1186/1472-6947-13-133 (PMC3913838; doi:10.1186/1472-6947-13-133)
Supplement: Additional file 5: Table S5 — Excluded non-randomized controlled studies (non-R,CTs) citations. [file 1472-6947-13-133-S5.pdf]

Table S7

Included randomized controlled trials (RCTs) citations

n = 18

**Ansari 2003**	Ansari M, Shlipak MG, Heidenreich PA, Van Ostaeyen D, Pohl EC, Browner WS, Massie BM: Improving guideline adherence: a randomized trial evaluating strategies to increase beta-blocker use in heart failure. *Circulation* 2003, 107:2799-2804.
**Berner 2006**	Berner ES, Houston TK, Ray MN, Allison JJ, Heudebert GR, Chatham WW, Kennedy JI, Jr., Glandon GL, Norton PA, Crawford MA et al.: Improving ambulatory prescribing safety with a handheld decision support system: a randomized controlled trial. *J Am Med Inform Assoc* 2006, 13:171-179.
**Dainty 2011**	Dainty KN, Adhikari NKJ, Kiss A, Quan S, Zwarenstein M: Electronic prescribing in an ambulatory care setting: a cluster randomized trial. *J Eval Clin Pract* 2011 Mar 18. [Epub ahead of print]. DOI: 10.1111/j.1365-2753.2011.01657.x.
**Feldstein 2006**	Feldstein AC, Smith DH, Perrin N, Yang X, Simon SR, Krall M, Sittig DF, Ditmer D, Platt R, Soumerai SB: Reducing warfarin medication interactions: an interrupted time series evaluation. *Arch Intern Med* 2006, 166:1009-1015.
**Fitzmaurice 1996**	Fitzmaurice DA, Hobbs FD, Murray ET, Bradley CP, Holder R: Evaluation of computerized decision support for oral anticoagulation management based in primary care. *Br J Gen Pract* 1996, 46:533-535.
**Fitzmaurice 2000**	Fitzmaurice DA, Hobbs FDR, Murray ET, Holder RL, Allan TF, Rose PE: Oral anticoagulation management in primary care with the use of computerized decision support and near-patient testing: A randomized, controlled trial. *Arch Intern Med* 2000, 160:2343-2348.
**Fortuna 2009**	Fortuna RJ, Zhang F, Ross-Degnan D, Campion FX, Finkelstein JA, Kotch JB, Feldstein AC, Smith DH, Simon SR: Reducing the prescribing of heavily marketed medications: a randomized controlled trial. *J Gen Intern Med* 2009, 24:897-903.
**Holt 2010**	Holt TA, Thorogood M, Griffiths F, Munday S, Friede T, Stables D: Automated electronic reminders to facilitate primary cardiovascular disease prevention: randomised controlled trial. *Br J Gen Pract* 2010, 60:251-256.
**McCowan 2001**	McCowan C, Neville RG, Ricketts IW, Warner FC, Hoskins G, Thomas GE: Lessons from a randomized controlled trial designed to evaluate computer decision support software to improve the management of asthma. *Med Inform Internet Med* 2001, 26:191-201.
**Montgomery 2000**	Montgomery AA, Fahey T, Peters TJ, MacIntosh C, Sharp DJ: Evaluation of computer based clinical decision support system and risk chart for management of hypertension in primary care: randomised controlled trial. *BMJ* 2000, 320:686-690.
**Poller 2009**	Poller L, Keown M, Ibrahim S, Lowe G, Moia M, Turpie AG, Roberts C, Van Den Besselaar AMHP, Van Der Meer FJM, Tripodi A et al.: A multicentre randomised assessment of the DAWN AC computer-assisted oral anticoagulant dosage program. *Thromb Haemost* 2009, 101:487-494.
**Tamblyn**	Tamblyn R, Huang A, Perreault R, Jacques A, Roy D, Hanley J, McLeod P, Laprise R:

**2003**	The medical office of the 21st century (MOXXI): effectiveness of computerized decision-making support in reducing inappropriate prescribing in primary care. *CMAJ* 2003, 169:549-556.
**Tamblyn 2012**	Tamblyn R, Eguale T, Buckeridge DL, Huang A, Hanley J, Reidel K, Shi S, Winslade N: The effectiveness of a new generation of computerized drug alerts in reducing the risk of injury from drug side effects: a cluster randomized trial. *J Am Med Inform Assoc* 2012, 19:635-643.
**Terrell 2009**	Terrell KM, Perkins AJ, Dexter PR, Hui SL, Callahan CM, Miller DK: Computerized decision support to reduce potentially inappropriate prescribing to older emergency department patients: a randomized, controlled trial. *J Am Geriatr Soc* 2009, 57:1388-1394.
**Terrell 2010**	Terrell KM, Perkins AJ, Hui SL, Callahan CM, Dexter PR, Miller DK: Computerized decision support for medication dosing in renal insufficiency: a randomized, controlled trial. *Ann Emerg Med* 2010, 56:623-629
**Tierney 2003**	Tierney WM, Overhage JM, Murray MD, Harris LE, Zhou XH, Eckert GJ, Smith FE, Nienaber N, McDonald CJ, Wolinsky FD: Effects of computerized guidelines for managing heart disease in primary care. *J Gen Intern Med* 2003, 18:967-976.
**Tierney 2005**	Tierney WM, Overhage JM, Murray MD, Harris LE, Zhou XH, Eckert GJ, Smith FE, Nienaber N, McDonald CJ, Wolinsky FD: Can computer-generated evidence-based care suggestions enhance evidence-based management of asthma and chronic obstructive pulmonary disease? A randomized, controlled trial. *Health Serv Res* 2005, 40:477-497.
**Vadher 1997**	Vadher BD, Patterson DLH, Leaning M: Comparison of oral anticoagulant control by a nurse-practitioner using a computer decision-support system with that by clinicians. *Clin Lab Haematol* 1997, 19:203-207.
