# Supplementary material for: Risks to patient safety associated with implementation of electronic applications for medication management in ambulatory care - a systematic review
Source: BMC Med Inform Decis Mak. 2013 Dec 5;13:133. doi: 10.1186/1472-6947-13-133 (PMC3913838; doi:10.1186/1472-6947-13-133)
Supplement: Additional file 6: Table S6 — Excluded observational studies citations. [file 1472-6947-13-133-S6.pdf]

Table S8

Included Non-randomized controlled studies citations

n = 5

**Abramson 2011**	Abramson EL, Malhotra S, Fischer K, Edward A, Pfoh ER, Osorio SN, Cheriff A, Kaushal R: Transitioning between electronic health records: Effects on ambulatory prescribing safety. *J Gen Intern Med* 2011, 26:868-874.
**Gandhi 2005**	Gandhi TK, Weingart SN, Seger AC, Borus J, Burdick E, Poon EG, Leape LL, Bates DW: Outpatient prescribing errors and the impact of computerized prescribing. *J Gen Intern Med* 2005, 20:837-841.
**Kirk 2005**	Kirk RC, Li-Meng GD, Packia J, Min KH, Ong BK: Computer calculated dose in paediatric prescribing. *Drug Saf* 2005, 28:817-824.
**Malone 2012**	Malone DC, Saverno KR: Evaluation of a wireless handheld medication management device in the prevention of drug-drug interactions in a medicaid population. *J Manage Care Pharm* 2012,18:33-45.
**Moniz 2011**	Moniz TT, Seger AC, Keohane CA, Seger DL, Bates DW, Rothschild JM: Addition of electronic prescription transmission to computerized prescriber order entry: Effect on dispensing errors in community pharmacies. *Am J Health-Syst Pharm* 2011, 68:158-163.
