# Supplementary material for: Risks to patient safety associated with implementation of electronic applications for medication management in ambulatory care - a systematic review
Source: BMC Med Inform Decis Mak. 2013 Dec 5;13:133. doi: 10.1186/1472-6947-13-133 (PMC3913838; doi:10.1186/1472-6947-13-133)
Supplement: Additional file 7: Table S7 — Included randomized controlled trials (RCTs) citations. [file 1472-6947-13-133-S7.pdf]

Table S9

Included observational studies citations

n = 16

**Åstrand 2009**	Astrand B, Montelius E, Petersson G, Ekedahl A: Assessment of ePrescription quality: an observational study at three mail-order pharmacies. *BMC Med Inf Decis Mak* 2009, 9:8.
**Bizovi 2002**	Bizovi KE, Beckley BE, McDade MC, Adams AL, Lowe RA, Zechnich AD, Hedges JR: The effect of computer-assisted prescription writing on emergency department prescription errors. *Acad Emerg Med* 2002, 9:1168-1175.
**Cafolla 2011**	Cafolla A, Melizzi R, Baldacci E, Pignoloni P, Dragoni F, Campanelli M, Caraccini R, Foa R: "Zeus" a new oral anticoagulant therapy dosing algorithm: A cohort study. *Thrombos Res* 2011, 128:325-330.
**Devine 2010**	Devine EB, Hansen RN, Wilson-Norton JL, Lawless NM, Fisk AW, Blough DK, Martin DP, Sullivan SD: The impact of computerized provider order entry on medication errors in a multispecialty group practice. *J Am Med Inform Assoc* 2010, 17:78-84.
**Ekedahl 2004**	Ekedahl A, Mansson N: Unclaimed prescriptions after automated prescription transmittals to pharmacies. *Pharm World Sci* 2004, 26:26-31.
**Ginzburg 2009**	Ginzburg R, Barr WB, Harris M, Munshi S: Effect of a weight-based prescribing method within an electronic health record on prescribing errors. *Am J Health-Syst Pharm* 2009, 66:2037-2041.
**Humphries 2007**	Humphries TL, Carroll N, Chester EA, Magid D, Rocho B: Evaluation of an electronic critical drug interaction program coupled with active pharmacist intervention. *Ann Pharmacother* 2007, 41:1979-1985.
**Kinnaird 2003**	Kinnaird D, Cox T, Wilson JP: Unclaimed prescriptions in a clinic with computerized prescriber order entry. *Am J Health-Syst Pharm* 2003, 60:1468-1470.
**Nanji 2011**	Nanji KC, Rothschild JM, Salzberg C, Keohane CA, Zigmont K, Devita J, Gandhi TK, Dalal AK, Bates DW, Poon EG: Errors associated with outpatient computerized prescribing systems. *J Am Med Inform Assoc* 2011, 18:767-773.
**Palchuk 2010**	Palchuk MB, Fang EA, Cygielnik JM, Labreche M, Shubina M, Ramelson HZ, Hamann C, Broverman C, Einbinder JS, Turchin A: An unintended consequence of electronic prescriptions: prevalence and impact of internal discrepancies. *J Am Med Inform Assoc* 2010, 17:472-476.
**Schwarz 2012**	Schwarz EB, Parisi SM, Handler SM, Koren G, Cohen ED, Shevchik GJ, Fischer GS: Clinical decision support to promote safe prescribing to women of reproductive age: A cluster-randomized trial. *J Gen Intern Med* 2012, 27:831-838.
**Steele 2005**	Steele AW, Eisert S, Witter J, Lyons P, Jones MHA, Gabow P, Ortiz E: The effect of automated alerts on provider ordering behavior in an outpatient setting. *Plos Medicine* 2005, 2:864-870.
**Varkey 2007**	Varkey P, Aponte P, Swanton C, Fischer D, Johnson SF, Brennan MD: The effect of computerized physician-order entry on outpatient prescription errors. *Manag Care Interface* 2007, 20:53-57.
**Volmer 2012**	Volmer D. Use of a generic protocol in documentation of prescription errors in Estonia, Norway and Sweden. *Pharmacy Practice* 2012, 10(2):72-77.

**Weingart 2003**	Weingart SN, Toth M, Sands DZ, Aronson MD, Davis RB, Phillips RS: Physicians' decisions to override computerized drug alerts in primary care. *Arch Intern Med* 2003, 163:2625-2631.
**Zillich 2008**	Zillich AJ, Shay K, Hyduke B, Emmendorfer TR, Mellow AM, Counsell SR, Supiano MA, Woodbridge P, Reeves P: Quality improvement toward decreasing high-risk medications for older veteran outpatients. *J Am Geriatr Soc* 2008, 56:1299-1305.
