# Supplementary material for: Risks to patient safety associated with implementation of electronic applications for medication management in ambulatory care - a systematic review
Source: BMC Med Inform Decis Mak. 2013 Dec 5;13:133. doi: 10.1186/1472-6947-13-133 (PMC3913838; doi:10.1186/1472-6947-13-133)
Supplement: Additional file 8: Table S8 — Included non-randomized controlled studies citations. [file 1472-6947-13-133-S8.pdf]

Table S10

Included randomized controlled studies (RCTs); Characteristics and outcomes (n= 18)

Estimates are reported for safety outcomes and adverse effects. For other outcomes, information is given if outcomes were significantly worse, significantly better, or no significant differences

	**Study Design**<br><br>RCT = randomized controlled trial<br><br>RCT- I = **individuals randomized**<br><br>RCT-C = RCT, cluster randomized<br><br>Unit(s) of randomization	**Setting**	**Intervention**<br><br>CDSS = computerised clinical decision support system<br><br>EHR: electronic health record<br><br>E-Rx: electronic prescribing<br><br>eGPP = electronically generated paper prescription	**Comparison**	***á priori* specified monitoring for safety outcomes or risks**<br><br>**Adverse events (AE) reported?**<br><br>**Risks to patient safety reported?**	**Process outcomes**	**For safety outcomes: Change in process outcomes consistent with (↑) or non-consistent with (↓) intent of intervention**<br><br>* : p calculated by reviewers<br><br>**For other outcomes:**<br><br>W: significantly worsened<br><br>B: significantly better<br><br>NS: no significant differences	**Patient outcomes**	**For safety outcomes: Change in patient outcomes consistent with (↑) or non-consistent with (↓) intent of intervention**<br><br>* : P calculated by reviewers<br><br>**For other outcomes:**<br><br>W: significantly worsened<br><br>B: significantly better<br><br>NS: no significant differences	**Uptake of e-intervention** non-use, override or rejection of CDSS suggestion:
1	**Ansari 2003**<br>- β-blocker use in heart failure<br><br>RCT-C<br><br>Patients randomized to prescriber<br><br>Prescriber randomized to intervention	Hospital out-patient clinic	CDSS in EHR<br><br>System initiated<br><br>eGPP<br><br>Computerized provider guideline based reminders (and patient letters advocating beta-blockers) to improve guideline adherence for β-blocker use in heart failure patients	Control and nurse facilitator	*á priori* specified- Information was collected on hospitalizations, emergency room visits, and deaths during follow-up as indicators of safety<br><br>AE reported: no<br><br>Risks to patient safety: See patient outcomes	% patients initiated or up-titrated and maintained on β-blockers<br><br>% patients initiated on β-blockers<br><br>% patients at target β-blockers doses at end of study<br><br>Time to reach target dose	W<br><br>W<br><br>W<br><br>W	Hospitalizations<br><br>ER visits during follow-up<br><br>Median hospitalizations or ER visits per patient<br><br>Deaths during follow-up	↑ 6%, p = 0.56 (*) 43% vs. 49%<br><br>↑ 1%, p = 1.0 (*) 9% vs. 10%<br><br>↓4% vs 2%<br><br>↑ 5%, p = 0.55 (*) 9% vs. 14%	33% of intervention group (physicians) started or adjusted medications compared to 67% in no CDSS groups (nurse and control)
2	**Berner 2006**<br>- NSAIDs	Hospital out-patient clinic	CDSS only<br><br>User initiated	Usual care	*á priori* specified- Yes, unsafe prescribing main	Mean proportion of patients per MD with unsafe prescribing	↑ effect size = 0.54, p < 0.05			not reported

	RCT- I<br><br>Prescriber randomized to intervention		Point-of-care hand-held risk assessment and treatment with NSAIDs		outcome<br><br>AE reported: no		0.23 vs. 0.45, p<0.05 This does not make sense.			
3	**Dainty 2011**<br>- Non-specific prescribing<br><br>RCT- C<br><br>Randomized by time period	Hospital out-patient clinic	E-Rx in conjunction with CDSS facility for computer-generated paper prescription<br><br>User initiated<br><br>Facility to transmit prescription to hospital or community pharmacy; pick-lists of medications for prescription writing	usual prescribing methods	*á priori* specified-no<br><br>AE reported: no	Total prescription error ratio<br><br><br>Pharmacy call-backs/week for clarification	↓ 0.1%, p = 0.91<br>6% vs. 5.9%<br><br><br>↓ 5.6%, p <0.001<br>1.89% vs. 1.45%			Low uptake of e-Rx, around 6%
4	**Feldstein 2006c**<br>-Warfarin medication interaction<br><br>RCT- C<br><br>Practices randomized to intervention	Doctor's office	CDSS in EHR<br><br>System initiated<br><br>Alerts for warfarin medication interactions	No alerts	*á priori* specified-yes; main outcome<br><br>AE reported: no	Total interacting prescription rate per month<br><br><br>Absolute Risk Reduction<br>(RRR) per month at 12 months	↑ slope -22.4/10000 patients,<br>P = 0.01<br><br><br>↑ 489.8<br>95%CI: -664.9 to – 314.7<br>(14.9%,95%CI:19.5% to -10.2%)			Not reported
5	**Fitzmaurice 1996**<br>- Oral anticoagulation therapy<br><br>RCT- I<br><br>Patient randomized to intervention	Doctor's office	CDSS<br><br>System initiated<br><br>Dosing of oral anticoagulants	Hospital clinic dosing	*á priori* specified-Yes, deaths and adverse events<br><br>AE reported:<br>Yes, one unconfirmed thrombotic episode in intervention vs. none in control. Otherwise, intervention overall had fewer adverse events.			INR control<br><br>Adverse events<br>- deaths<br>- thrombotic episodes<br><br>- hemorrhagic episodes	NS<br><br><br>↑ 1 vs. 2<br>↓ 1 (unconfirmed) vs. 2<br>↑ 3 (2 epistaxes; 1 bruising) vs. 5 (3 epistaxes; 1 gum bleed; 1 haematoma)	Not reported
6	**Fitzmaurice 2000**	Hospital out-	CDSS	Hospital clinic	*á priori* specified-			Results given for intervention		

	- Oral anticoagulation therapy<br><br>RCT- I<br><br>Patients randomized to intervention	patient clinic and doctor's office	System initiated<br><br>Dosing recommendations for patients taking oral anticoagulation therapy (Warfarin)	dosing	Yes;<br>adverse events, particularly hemorrhagic and thromboembolic episodes<br><br>AE reported: yes, see patient outcomes			(number of patients= 122) vs. intra-practice control (number of patients= 102)<br><br>Proportion of patients achieving individual INR targets<br><br>Percentage of time in INR range<br><br>Adverse events: thromboembolic episodes<br>- Deep venous thrombosis<br>- Transient ischemic attack<br>- Fatal CVA<br>- Non-fatal CVA (infarct)<br>- Saddle embolus<br>- Epistaxis<br><br>Death rates<br>- Stroke<br>- CHF<br>- Ischemic heart disease<br>- Left ventricular failure<br>. Renal failure<br>- Carcinoma	 <br><br>NS<br><br><br>B<br><br><br><br>↓ 1 vs. 0<br>↑ 0 vs. 1<br>↓ 1 vs. 0<br>↑ 0 vs. 1<br>↑ 0 vs. 1<br>↓ 1 vs. 0<br><br><br>↓ 1 vs. 0<br>↓ 1 vs. 0<br>0 vs 0<br>↑ 0 vs. 1<br>↑ 0 vs. 1<br>1 vs. 1	Not reported
7	**Fortuna 2009**<br>- Hypnotics for elderly patients<br><br>RCT- C<br><br>Practices randomized to intervention	Doctor's office	CDSS with EHR<br><br>System initiated<br><br>Alerts to support evidence based prescribing with and without physician-led educational sessions, to reduce prescribing of certain hypnotics	Usual care	*á priori* specified- Risk of prescribing certain hypnotics<br><br>AE reported: no	Ratio of relative risk of prescriptions for certain hypnotics<br><br>Changes in proportion of prescriptions for certain hypnotics	Ratio of adjusted RR (95% CI)<br>0.74(0.57 to 0.96)<br><br>↑ Adjusted RR (95% CI) 0.97 (0.82 to 1.14) vs. 1.31 (1.08 to 1.60)			Not reported
8	**Holt 2010**<br>- Risk alerts for CVD	Doctor's office	CDSS in EHR	No alerts	*á priori specified:*<br>Yes, main outcome			annual cardiovascular event rate	↑Rate ratio 0.96, p= 0.02 (95%	Not reported

	RCT- I<br><br>Patients randomized to intervention		System initiated<br><br>alerts of risk of coronary vascular disease		AE reported: no				C.I.0.85 to 1.10)	
9	**McCowan 2001**<br>- Asthma<br><br>RCT- C<br><br>Practices randomized to intervention	Doctor's office	CDSS<br><br>System initiated<br><br>e-delivery of guidelines for asthma	Usual care	*á priori* specified- Yes: hospital admissions, A&E attendances, outpatient consultation, acute exacerbations of asthma<br><br>AE reported: Yes, see patient outcomes	Level of maintenance prescribing	NS	Percent of patients who initiated an asthma consultation<br><br>Acute exacerbation of asthma<br><br>Need for acute prescription for oral corticosteroids<br><br>Need for emergency nebulisations<br><br>Admittance to hospital<br><br>Need to attend ER<br><br>Need to attend outpatient departments	↑ OR 0.59 (95% C.I. 0.37 to 0.95)<br>22% vs. 34%<br><br>↑ OR 0.43 (95% C.I. 0.21 to 0.85)<br>8% vs.1 7%<br><br>↑OR 0.42 (95% C.I. 0.14 to 1.29)<br>5% vs. 11%<br><br>↑ OR 0.13 (95% C.I. 0.01 to 0.91)<br>1% vs. 5%<br><br>↑ OR 0 (95% C.I. 0 to 3.44)<br>0% vs. 1%<br><br>↑ OR 0 (95% C.I 0 to 9.16)<br>0 vs. 1%<br><br>↑ OR 0.64 (95% C.I 0.09 to 3.38)<br>1% vs. 2%	Not reported
10	**Montgomery 2000**<br>- Hypertension<br><br>RCT- C<br><br>Practices randomized to intervention	Doctor's office	CDSS and user or system initiated EHR<br><br>CDSS system initiated<br><br>CDSS cardiac risk calculation and cardiovascular risk chart combined for appropriate prescribing for	Traditional prescribing<br>or<br>Risk Chart<br><br>Risk chart alone	*á priori* specified- Yes, high risk of cardiovascular event, see patient outcomes<br><br>AE reported: no, only proportion of patients with high risk			Proportion of patients with high risk of cardiovascular event<br>- vs Traditional dosing<br><br>- vs Risk chart only	↓ OR = 1.7 (95% C.I 0.7 to 3.9) p = 0.43<br><br>↓ OR = 2.3 (95% C.I 1.1 to 4.8) p = 0.02	Not reported

			hypertension							
11	**Poller 2009**<br>- Oral anticoagulation therapy<br><br>RCT- I<br><br>Patients randomized to intervention	Hospital out-patient clinic	CDSS<br><br>System initiated<br><br>Computer-generated dosing of anti-coagulant therapy	Traditional dosing	*á priori* specified- Major bleeds, thrombotic events, death.<br><br>AE reported:<br>Higher risk (NS) for major bleeds (1.4 versus 0.9 events per 100 patient years) with computer-assisted anticoagulant dosing			Overall time in target range<br><br>Events per 1000 patient years<br><br>- Major bleeds<br><br>- Minor bleeds<br><br>- Thrombotic events<br><br>- Deaths	B<br><br>↓ 1.4 vs. 0.9 (p = 0.404)*<br><br>↑ 2.5 vs. 2.7 (p = 0.8896)*<br><br>↑ 0.9 vs. 1.4 9 (p = 0.404)*<br><br>0.8 vs. 0.8<br><br>* 2-tailed chi-squared with Yate's correction	27.2% of computer dosing advice was not taken.
12	**Tamblyn 2003**<br>- Elderly patients<br><br>RCT- I<br><br>Prescribers randomized to intervention	Doctor's offices	CDSS with EHR<br><br>System initiated<br><br>Alerts identifying potential prescribing problems, consequences and alternative suggestions to reduce inappropriate prescribing in elderly patients for selected drugs	No CDSS	*á priori* specified-no<br><br>AE reported: no	Initiation rate of inappropriate prescribing<br><br>- Overall<br><br>- Drug - disease contraindications<br><br>- Drug-age contraindication<br><br>- Excessive duration of therapy<br><br>- Therapeutic duplication<br><br>- Drug interaction<br><br>- Discontinuation of inappropriate prescribing<br><br>Discontinuation of inappropriate prescribing<br><br>- Overall<br><br>- For drug-disease contraindication<br><br>- For drug-age contraindication	RR (95% C.I.)<br><br>↑ 0.8 (0.69 to 0.98)<br><br>↑ 0.89 (0.72 to 1.10)<br><br>↑ 0.77 (0.59 to 1.00)<br><br>↑ 0.78 (0.61 to 0.99)<br><br>↑ 0.78 (0.61 to 0.99)<br><br>↓1.12 (0.68 to 1.87)<br><br>↓1.06 (0.89 to 1.26)<br><br>RR (95% C.I.)<br><br>↑ 1.06 (0.89 to 1.26)<br><br>↑ 1.08 (0.85 to 1.36)<br><br>↓0.94 (0.79 to 1.1)			Not clear

						- For excessive duration of therapy<br><br>- For therapeutic duplication<br><br>- For drug interaction	1.00 (0.77 to 1.29)<br><br>↓0.94 (0.54 to 1.51)<br><br>↑ 1.33 (0.90 to 1.95)			
13	**Tamblyn 2012**<br>- Psychotropics for elderly patients<br><br>RCT- I)<br><br>Prescribers randomized to intervention	Doctor's office	CDSS: patient-specific risk of injury alert upon prescribing of psychotropics to elderly patients<br><br>System initiated	No alert	*á priori specified:* yes, main outcome "risk of injury"<br><br>AE reported: no	Physician's response to the injury risk alert (observational, non-comparative)<br><br>Changes in the use and dose of psychotropic medications (observational, non-comparative)	83.3%<br><br>41%	Reduction in risk of injury per 1000 patients	↑1.7 (95% C.I. 0.2 to 3.2; p= 0.02)	yes, process outcomes: 41% alerts not followed
14	**Terrell 2009**<br>- Elderly patients<br><br>RCT- I<br><br>Prescribers randomized to intervention	Emergency room	CDSS with EHR-CPOE—emergency room)<br><br>System initiated<br><br>CDSS triggered upon tentatively inappropriate prescribing for patients over 64 to reduce potentially inappropriate prescribing	Usual care	*á priori* specified: no<br><br>AE reported: no	Visits with an inappropriate medication prescription<br><br>Prescriptions that were inappropriate	↑ 2.6% vs. 3.9%, p = 0.02, OR: 0.55 (C.I. 0.34 to 0.89)<br><br>↑ 3.4% vs. 5.4%, p = 0.006, OR: 0.59 (C.I. 0.41 to 0.85)			57% of intervention suggestions rejected
15	**Terrell 2010**<br>- Renal insufficiency<br><br>RCT- I<br><br>Prescribers randomized to intervention	Emergency room	CDSS integrated in EHR<br><br>System initiated<br><br>To facilitate appropriate dosing of targeted medications for adult patients with renal insufficiency	Usual care	*á priori* specified- excessive dosing<br><br>AE reported: no	Rate of excessive prescribing of targeted medications overall	↑ Effect Size 31% (95% C.I. 14% to 49%)<br>43% vs. 74%, p = 0.0012*			Prescribers did not follow suggestions for 43% of prescriptions

			being discharged home from the ER							
16	**Tierney 2003**<br>- Heart failure and ischemic heart disease<br><br>RCT- C<br><br>Time periods randomized to intervention	Doctor's office	CDSS withEHR<br><br>System initiated<br><br>Evidence-based medication management suggestions for patients with heart failure and ischemic heart disease	Usual care	*á priori* specified: Yes, clinic visits, hospitalizations and other adverse events<br><br>AE reported: not other than as prespecified patient outcomes	Adherence to suggestion to<br><br>- start or increase an ACE inhibitor<br><br>- prescribe pneumococcal vaccination<br><br>-start or increase a β-blocker<br><br>-start low-dose aspirin<br><br>-start or increase a diuretic<br><br>-start or increase a long-acting nitrate<br><br>-start an antihyperlipidemic drug<br><br>-start or increase a calcium channel blocker:	 <br>NS<br><br>NS<br><br>NS<br><br>NS<br><br>NS<br><br>NS<br><br>NS<br><br>NS	Adverse events<br><br>- Number of all emergency department visits<br><br>- Number of heart disease specific emergency department visits<br><br>- Number of all hospitalizations<br><br>- Number of heart disease specific hospitalizations	mean/person<br><br>↓ 1.1 (SD 1.9) vs. 1.0 (SD 1.7)<br><br>0.2 (SD 0.4) vs. 0.2 (SD 0.5)<br><br>0.2 (SD 0.4) vs. 0.2 (SD 0.5)<br><br>0.2 (SD 0.45) vs. 0.2 (SD 0.6)	Not reported
17	**Tierney 2005**<br>- Medication and vaccination suggestions for asthma and COPD<br><br>RCT- C<br><br>Time periods randomized to intervention	Hospital out-patient clinic	CDSS with EHR<br><br>System initiated<br><br>Patient-specific medication and vaccination suggestions based on evidence-based guidelines for asthma and COPD	No suggestions	*á priori* specified: Yes, clinic visits, hospitalizations and other adverse events<br><br>AE reported: not other than as prespecified patient outcomes<br><br>.	Compliance with medication treatment guidelines for medication to<br><br>- prescribe influenza vaccination<br><br>- prescribe pneumococcal vaccination<br><br>- Start ipratropium<br><br>- Start inhaled β-agonist<br><br>- Increase/ decrease theophylline dose<br><br>- Stop ipratropium	 <br>NS<br><br>NS<br><br>NS<br><br>NS<br><br>NS<br><br>NS	Number of all emergency department visits<br><br>Number of emergency department visits for reactive airways disease<br><br>Number of all hospitalizations<br><br>Number of hospitalizations for reactive airways disease	1.4 (SD 1.7) vs. 1.4 (SD 1.59)<br><br>0.3 (SD 0.7) vs. 0.3 (SD 0.8)<br><br>↑ 0.5 (SD 1.6) vs. 0.4 (SD 0.8)<br><br>↓ 0.5 (SD 0.5) vs. 0.1 (SD 0.3)	Physicians did not adhere to 68% of suggestions overall.

						- Start inhaled corticosteroid<br><br>- Start oral corticosteroid	NS<br><br>NS			
18	**Vadher 1997**<br>- Oral anticoagulant control<br><br>RCT- I<br><br>Patients randomized to intervention	Hospital out-patient clinic	CDSS for nurses<br><br>System initiated<br><br>Oral anticoagulant control by nurse using CDSS	Trainee doctors without CDSS	*á priori* specified- YES, thrombosis, hemorrhage<br><br>AE reported: Yes, patient outcomes	Adherence to guidelines for<br><br>- INR range group (2-3)<br><br>- INR range group (3-4.5)	B<br><br>NS	Time (days per 100 patient days of treatment) in INR therapeutic range<br><br>- range group (2-3)<br><br>- range group (3-4.5)<br><br>Thrombotic events<br><br>Hemorrhagic events	B<br><br>NS<br><br>intervention: 1 vs. control : 2<br><br>intervention: 2 vs. control : 1	Adherence to guideline in lower INR group intervention 88% vs. control 60%; P = <0.01<br><br>Adherence to guideline in higher INR group intervention 67% vs. control 73%; P =0.18
