# Supplementary material for: Risks to patient safety associated with implementation of electronic applications for medication management in ambulatory care - a systematic review
Source: BMC Med Inform Decis Mak. 2013 Dec 5;13:133. doi: 10.1186/1472-6947-13-133 (PMC3913838; doi:10.1186/1472-6947-13-133)
Supplement: Additional file 9: Table S9 — Included observational studies citations. [file 1472-6947-13-133-S9.pdf]

Table S11 Quality assessment of included RCTs									
**Method of protection against bias**	Allocation concealment with explicit randomization	Follow-up of appropriate unit included (practice &/or professional &/or patient)	Blinded assessment of primary outcome(s)	Baseline measurement of outcome variables	Reliability of reported primary outcome measures	Protection against contamination	All outcomes reported	Declaration of interests and funding	Randomization and adjustment for clustering when indicated (NN = not necessary)
**Type of bias**	Selection bias	Exclusion bias	Detection bias	Selection bias	Detection bias	Performance bias	Reporting bias	Risk of bias due to funding or conflict of interest	Threat to reliability of variance estimate
Ansari 2003	low risk of bias	low risk of bias	low risk of bias	unclear risk of bias	unclear risk of bias	low risk of bias	low risk of bias	low risk of bias	RCT cluster randomized<br><br>Patients randomized to prescriber<br><br>Prescriber randomized to intervention<br><br>Patient-outcomes clustered under prescriber, adjusted for in analysis

Berner 2006	low risk of bias	low risk of bias	low risk of bias	low risk of bias	unclear risk of bias	unclear risk of bias	low risk of bias	low risk of bias	RCT (individual randomized)<br><br>Prescriber randomized to intervention<br><br>NN
Dainty 2011	low risk of bias	unclear risk of bias	low risk of bias	unclear risk of bias	unclear risk of bias	low risk of bias	low risk of bias	low risk of bias	RCT cluster randomized<br><br>Randomized by time period<br><br>NN: Planned adjustment for patient clustering under prescriber not necessary because number of prescribing errors per prescriber was low
Feldstein 2006	low risk of bias	unclear risk of bias	low risk of bias	unclear risk of bias	unclear risk of bias	low risk of bias	low risk of bias	low risk of bias	RCT cluster randomized<br><br>Practices randomized to intervention<br><br>Correct unit of analysis used to allow for effects of clustering of physicians under clinic
Fitzmaurice 1996	unclear risk of bias	unclear risk of bias	low risk of bias	unclear risk of bias	unclear risk of bias	unclear risk of bias	low risk of bias	Risk: funding source not found	RCT (individual-randomized)<br><br>Patient randomized to intervention

									Patient-outcomes naturally clustered under prescriber, **-not adjusted for in analysis**
Fitzmaurice 2000	low risk of bias	low risk of bias	low risk of bias	unclear risk of bias	unclear risk of bias	low risk of bias	low risk of bias	low risk of bias	RCT (individual-randomized)<br><br>Patients randomized to intervention<br><br>Patient-outcomes naturally clustered under prescriber, **-not adjusted for in analysis**
Fortuna 2009	low risk of bias	low risk of bias	low risk of bias	unclear risk of bias	low risk of bias	low risk of bias	low risk of bias	low risk of bias	RCT cluster randomized<br><br>Practices randomized to intervention<br><br>Adjusted in analysis for clustering of prescriber outcomes under practice
Holt 2010	low risk of bias	low risk of bias	low risk of bias	Unclear risk of bias	low risk of bias	Unclear risk of bias	low risk of bias	low risk of bias	RCT (individual-randomized)<br><br>Patient randomized to intervention<br><br>Patient-outcomes naturally clustered under prescriber,

									**-not adjusted for in analysis**
McCowan 2001	low risk of bias	not low risk of bias	low risk of bias	unclear risk of bias	unclear risk of bias	low risk of bias	low risk of bias	Risk : some industry funding	RCT cluster randomized<br><br>Practices randomized to intervention<br><br>Adjustment in analysis for the effects of clustering of prescriber under practice on prescriber outcomes was **not reported**<br><br>Patient-outcomes clustered under practice, - not adjusted for in analysis
Montgomery 2000	low risk of bias	low risk of bias	low risk of bias	low risk of bias	unclear risk of bias	low risk of bias	low risk of bias	low risk of bias	RCT cluster randomized<br><br>Practices randomized to intervention<br><br>Patient-outcomes clustered under practice, adjusted for in analysis
Poller 2009	unclear risk of bias	low risk of bias	low risk of bias	low risk of bias	low risk of bias	low risk of bias	low risk of bias	low risk of bias	RCT (individual-randomized)<br><br>Patients randomized to intervention<br><br>Patient-outcomes naturally clustered

									under prescriber or practice<br>-**not adjusted for in analysis**
Tamblyn 2003	unclear risk of bias	low risk of bias	low risk of bias	low risk of bias	low risk of bias	low risk of bias	low risk of bias	low risk of bias	RCT (individual-randomized)<br><br>Prescribers randomized to intervention<br><br>Adjusted in analysis for possible effects of natural clustering of patients under prescriber on prescriber process outcomes
Tamblyn 2012	low risk of bias	low risk of bias	low risk of bias	low risk of bias	low risk of bias	Unclear risk of bias	low risk of bias	low risk of bias	RCT (individual-randomized)<br><br>Prescribers randomized to intervention<br><br>Adjusted in analysis for possible effects of natural clustering of patients under prescriber on prescriber process outcomes
Terrell 2009	low risk of bias	low risk of bias	low risk of bias	unclear risk of bias	low risk of bias	low risk of bias	low risk of bias	low risk of bias	RCT (individual-randomized)<br><br>Prescribers randomized to intervention<br><br>Adjusted in

									analysis for possible effects of natural clustering of patients under prescriber on prescriber process outcomes
Terrell 2010	low risk of bias	low risk of bias	low risk of bias	unclear risk of bias	low risk of bias	Unclear risk of bias	low risk of bias	low risk of bias	RCT (individual-randomized)<br><br>Prescribers randomized to intervention<br><br>Adjusted in analysis for possible effects of natural clustering of patients under prescriber on prescriber process outcomes
Tierney 2003	low risk of bias	low risk of bias	low risk of bias	unclear risk of bias	unclear risk of bias	low risk of bias	low risk of bias	low risk of bias	RCT cluster randomized<br><br>Time periods randomized to intervention<br><br>Adjusted in analysis for clustering of patient outcomes clustered under prescriber
Tierney 2005	low risk of bias	low risk of bias	low risk of bias	unclear risk of bias	low risk of bias	low risk of bias	low risk of bias	low risk of bias	RCT cluster randomized<br><br>Time periods randomized to intervention<br><br>Adjusted in analysis for

									clustering of patient outcomes clustered under prescriber
Vadher 1997	low risk of bias	unclear risk of bias	low risk of bias	low risk of bias: for median durations of anticoagulation at baseline statistically different; Otherwise, unclear risk of bias for baseline prescribing behavior	unclear risk of bias	low risk of bias	low risk of bias	low risk of bias	RCT (individual-randomized)<br><br>Patients randomized to intervention<br><br>Patient-outcomes naturally clustered under prescriber or practice<br>-**not adjusted for in analysis**
