# Supplementary material for: Risks to patient safety associated with implementation of electronic applications for medication management in ambulatory care - a systematic review
Source: BMC Med Inform Decis Mak. 2013 Dec 5;13:133. doi: 10.1186/1472-6947-13-133 (PMC3913838; doi:10.1186/1472-6947-13-133)
Supplement: Additional file 10: Table S10 — Included randomized controlled studies (RCTs); characteristics and outcomes. [file 1472-6947-13-133-S10.pdf]

Table S12

Included non-randomized controlled trials (non-R,CTs); characteristics and outcomes (n = 8)

	**Study**<br><br>**Design**	**Setting**<br><br>Hospital out-patient clinic<br><br>Doctor's office<br><br>Hospital out-patient clinic and doctor's office<br><br>Emergency room	**Intervention**<br><br>CDSS = computerised clinical decision support system;<br><br>EHR: electronic health record;<br><br>E-Rx; electronic prescribing<br><br>eGPP = electronically generated paper prescription	**Comparison**	**Process outcomes**	**Change in process outcomes in intended direction**<br><br>W: significantly worsened<br><br>B: significantly better<br><br>NS: no significant differences<br>**Change in process outcomes consistent with (↑) or non-consistent with (↓) intent of intervention**	**Patient outcomes**	**Change in patient outcome**<br><br>W: significantly worsened<br><br>B: significantly better<br><br>NS: no significant differences	**Harms or risk of harms *á priori* specified-?**<br><br>**AE reported**<br><br>**Risks to patient safety reported**
1	**Abramson 2011**<br><br>controlled before and after	Doctor's office	CDSS in EHR with e-Rx capability	no CDSS in EHR	Rate of prescribing error	NS			*á priori* specified-main outcome<br><br>AE reported-no<br><br>Risks to patient safety reported-no

2	**Gandhi 2005**<br><br>prospective cohort	Hospital out-patient clinic and doctor's office	Computer-generation of paper prescriptions	handwritten prescribing	medication prescribing errors<br><br>potential adverse drug events	NS<br><br>NS			*á priori* specified-yes, main outcome<br><br>AE reported-no<br><br>Risks to patient safety reported: -yes, as main outcomes
3	**Kirk 2005**<br><br>prospective cohort	Hospital out-patient clinic	CDSS in EHR<br><br>User initiated<br><br>Dose calculation for pediatric patients: paracetamol and promethasine	no CDSS	Rates of prescribing errors	↑B			*á priori* specified-main outcome<br><br>AE reported-no<br><br>Risks to patient safety reported-yes, all errors in CDSS intervention group were due to MD override
4	**Malone 2012**<br><br>before and after	Doctor's office	CDSS in palm pilot<br><br>User initiated<br><br>To reduce likelihood of prescribing at least 1 potential drug-drug interaction	no CDSS	probability of prescribing drug-drug interaction	NS			*á priori* specified-no<br><br>AE reported-no<br><br>Risks to patient safety reported-no
5	**Moniz 2011**<br><br>before and after	Hospital out-patient clinic	e-Prescribing<br><br>Prescriptions transmitted to pharmacy electronically (e-prescribing)	paper prescriptions	Prescribing error rates	↑ Error rate for e-prescriptions declined significantly while non-eRx errors did not change.			*á priori* specified-no<br><br>AE reported-no<br><br>Risks to

									patient safety reported- no
