# Supplementary material for: Risks to patient safety associated with implementation of electronic applications for medication management in ambulatory care - a systematic review
Source: BMC Med Inform Decis Mak. 2013 Dec 5;13:133. doi: 10.1186/1472-6947-13-133 (PMC3913838; doi:10.1186/1472-6947-13-133)
Supplement: Additional file 11: Table S11 — Quality assessment of included RCTs. [file 1472-6947-13-133-S11.pdf]

Table S13

Included observational studies; characteristics and outcomes (n = 16)

	Study<br><br>Design	Setting<br><br>Hospital out-patient clinic<br><br>Doctor's office<br><br>Hospital out-patient clinic and doctor's office<br><br>Emergency room<br><br>Other, home visits	Intervention	Comparison<br><br>CDSS = computerised clinical decision support system;<br><br>EHR: electronic health record;<br><br>E-Rx; electronic prescribing<br><br>eGPP = electronically generated paper prescription	Process outcomes	Change in process outcomes in intended direction<br><br>Change in process outcomes consistent with (↑) or non-consistent with (↓) intent of intervention<br><br>W: significantly worsened<br><br>B: significantly better<br><br>NS: no significant differences<br><br>or estimate<br><br>or text	Patient outcomes	Change in patient outcome<br><br>Change in patient outcomes consistent with (↑) or non-consistent with (↓) intent of intervention<br><br>W: significantly worsened<br><br>B: significantly better<br><br>NS: no significant differences<br><br>or estimate<br><br>or text	Harms or risk of harms *á priori* specified<br><br>AE reported<br><br>Risks to patient safety reported
1	**Astrand 2009**	Ambulatory, not clear or not specified	e-prescribing	non e-prescribing	Number and frequencies of prescriptions where pharmacists needed	↓ RR of 1.7 in disfavor of e-prescribing			*á priori* specified-no<br><br>AE reported-no

	prospective observational comparison				clarification from prescriber for incorrect or incomplete prescribing, adverse events risks and drug-drug interaction risks				Risks to patient safety reported-yes, main outcome. Faulty dosage and directions for use could have caused patient harm if not intercepted by pharmacist.
2	**Bizovi 2002**<br><br>non-controlled before and after<br><br>Retrospective cohort	Emergency room	Computer generation of paper prescription	pre-intervention; handwritten prescriptions	Total potential adverse drug events (RR)	B			*á priori* specified-Yes: main outcome<br><br>AE reported- no<br><br>Risks to patient safety reported-Yes, faulty programming
3	**Cafolla 2011**<br><br>non-controlled before and after<br><br>Retrospective cohort	ambulatory, not specified	CDSS in EHR<br><br>System initiated<br><br>computer dosing of oral anticoagulants	pre-intervention; no CDSS			% of time spent in therapeutic range<br><br>major and minor bleeds and deaths<br><br>thromboembolic complications	B<br><br><br>NS<br><br>NS	*á priori* specified-Yes: major and minor bleeds; thromoembolic complications<br><br>AE reported- no<br><br>Risks to patient safety reported-no
4	**Devine 2010**<br><br>non-controlled before and after<br><br>Retrospective cohort	Doctor's office	CDSS alerts embedded in EHR with options for printing or faxing of prescriptions system initiated<br><br>System initiated	pre-intervention; no CDSS alerts	prescribing errors<br><br>severity of prescribing errors	B<br><br>B	Adverse drug events	post intervention 0.1% (n=5) vs pre-intervention 0.2% (n=8)	*á priori* specified-yes, main outcome<br><br>AE reported-Adverse drug events post intervention 0.1% (n=5) vs pre-intervention 0.2% (n=8)<br><br>Risks to patient

									safety reported- Lower rates of all error types with CDSS
5	**Ekedahl 2004**<br><br>retrospective cross-sectional	Hospital out-patient clinic and doctor's office	e-prescribing	none			Patient primary non- compliance i.e. proportion of unclaimed e-prescriptions	Primary non-compliance: 2.3% Highest numbers of unclaimed e-Rxs were for respiratory, nervous system and musculo-skeletal system drugs.	*á priori* specified-no<br><br>AE reported? no<br><br>Risks to patient safety reported- yes, main outcome
6	**Ginzburg 2009**<br><br>non-controlled before and after<br><br>retrospective cohort	Hospital out-patient clinic	CDSS in EHR<br><br>system initiated<br><br>Weight-based prescribing method pediatric acetaminophen or ibuprofen	pre-intervention; no CDSS	prescribing errors<br><br>strength over-dosing errors	B<br><br>B			*á priori* specified- yes, main outcome<br><br>AE reported - no<br><br>Risks to patient safety reported – yes, prescribing errors
7	**Humphries 2007**<br><br>interrupted time-series without control	Doctor's office	CDSS in EHR with e-prescribing<br><br>System initiated<br><br>Alerts for interacting crug-pairs	pre-intervention; no CDSS alerts	Proportion of instances of co-dispensing interacting drugs pairs	B			*á priori* specified- yes, main outcome<br><br>AE reported - no<br><br>Risks to patient safety reported - no
8	**Kinnaird 2003**<br><br>retrospective cohort; no comparison group	Hospital out-patient clinic	e-prescribing	none			patients not retrieving prescribed medications at pharmacy	2.8% of prescriptions not claimed<br><br>33% of unclaimed prescriptions considered essential	*á priori* specified- yes, main outcome<br><br>AE reported- no<br><br>Risks to patient safety reported- main outcome
9	**Nanji 2011**<br><br>retrospective cohort	ambulatory, not specified	Paper or e-prescriptions generated from EHR	none	% prescriptions with medication errors<br><br>% prescriptions with potential	11.7%<br><br>4.2%			*á priori* specified-yes, main outcomes<br><br>AE reported-no

					ADEs				Risks to patient safety reported- yes, main outcomes
10	**Palchuk 2010**<br><br>retrospective survey	Doctor's office	e-prescribing in EHR	none	Discrepancies between free text fields and check-off fields in individual Rxs	Rx with ≥1 discrepancy: 5.8%, of which 16.8% could lead to hospital admission or death<br><br>Discrepancy rate of high risk for ADE medications = 24%			*á priori* specified-yes, main outcome<br><br>AE reported-no<br><br>Risks to patient safety reported- High rate of discrepancies that could lead to high risk ADEs
11	Schwarz 2012<br><br>RCT; Comparison of two versions of same CDSS program	Hospital out-patient clinic and doctor's office	CDSS in EHR<br><br>Simple alerts vs. multifaceted alerts regarding prescribing safety for women who might be pregnant	no usual care comparison	risky prescribing; i.e. prescriptions of teratogenic medications	NS			*á priori* specified- yes, main outcome<br><br>AE reported-no<br><br>Risks to patient safety reported- no
12	Steele 2005<br><br>non-controlled before and after<br><br>retrospective cohort	Hospital out-patient clinic	CDSS in EHR<br><br>System initiated<br><br>Alerts for drug-laboratory interaction	pre-intervention; no CDSS alerts	Probability of adverse drug events<br><br>Number of medication orders not completed after any alert display<br><br>Number of medication orders not completed after alert display for abnormal laboratory values	NS<br><br>NS<br><br>B			*á priori* specified- yes, main outcome<br><br>AE reported - no<br><br>Risks to patient safety reported - no

13	Varkey 2007<br><br>retrospective survey	Hospital out-patient clinic	CDSS with computer generated paper prescriptions<br><br>CDSS initiation not clear	Handwritten prescriptions	pharmacist intercepted Rx with errors	significant decrease in error (4.9% vs 7.4%, p= .0048)			*á priori* specified-no<br><br>AE reported-no<br><br>Risks to patient safety reported-no
14	Volmer 2012<br><br>observational	ambulatory, not specified	e-Rx or computer-generated paper prescription	Manually written prescriptions	Comparison of frequency of error between e-Rx, PC generated paper Rx and handwritten Rx	Less errors for e-Rx compared to computer-generated or manually written prescriptions			*á priori* specified-no<br><br>AE reported-no<br><br>Risks to patient safety reported-no
15	Weingart 2003<br><br>retrospective series	Doctor's office	CDSS in EHR<br><br>System initiated<br><br>Allergy and drug interaction alerts	none	Decision to override alerts	override of 91.2% of drug allergy alerts<br><br>89.4% level 1 drug interaction alerts<br><br>96.3% of level 2 drug interaction alerts<br><br>85.4% of level 3 drug interaction alerts.	adverse drug events	Three interacting drug perscriptions resulting in<br>- 1supratherapeutic anticoagulation<br>- one epistaxis<br>- itching triggered by drug interaction	*á priori* specified- yes, main outcome<br><br>AE reported- 3 ADEs occurred in prescribing non-compliant with alerts<br><br>Risks to patient safety reported-yes, overriding of prescribing alerts
16	Zillich 2008<br><br>Non-controlled before and after<br><br>Retrospective cohort	Hospital out-patient clinic	CDSS in EHR<br><br>System initiated<br><br>Drug-specific alerts for prescribing for elderly patients	pre-intervention; no CDSS alerts	Patients with ≥1 high-risk medication	B			*á priori* specified-yes, main outcome<br><br>AE reported-no<br><br>Risks to patient safety reported-yes, overriding of prescribing alerts
